# Adult ileocecal intussusception as an unusual presentation of ascending colon adenocarcinoma: a case report from Sudan

**DOI:** 10.1093/jscr/rjae337

**Published:** 2024-05-28

**Authors:** Nadir Ali Hilal, Ahmed Rafei

**Affiliations:** Colorectal Surgery, National Center for Gastrointestinal and Liver Diseases, PO Box 12810, Khartoum, Sudan; Department of Research, National Center for Gastrointestinal and Liver Diseases, PO Box 12810, Khartoum, Sudan

**Keywords:** colonic intussusception, colon cancer, familial colon cancer, case report, Sudan

## Abstract

Adult colonic intussusception, is a rare entity that is typically associated with underlying organic pathologies, notably colorectal tumors, unlike pediatric cases, which are mostly idiopathic. We present a unique case of a 42-year-old female with ascending colon adenocarcinoma, where ileocecal intussusception served as the initial clinical manifestation. The patient’s non-specific symptoms, familial history of colon cancer and subsequent diagnostic evaluations underscore the importance of considering malignancy in such presentations. Successful laparoscopic right hemicolectomy resolved the intussusception. This case, which is the first case to be reported in Sudan, highlights the clinical complexities of adult colonic intussusception, emphasizing the need for a heightened index of suspicion for underlying malignancy and the significance of timely surgical intervention. Furthermore, the challenges encountered in resource-limited settings underscore the necessity for genetic testing to guide familial screenings and identify hereditary factors contributing to colon cancer, providing valuable insights for clinicians managing similar cases.

## Introduction

The term ‘intussusception’ refers to the invagination of one intestinal segment into the neighboring segment, causing bowel obstruction and potential intestinal ischemia [1]. Colonic intussusception that manifests in adulthood is extremely rare, occurring in less than 1 in 1300 abdominal surgeries. This occurrence accounts for 1% of cases of small bowel obstruction and even less frequently for large bowel obstruction [2].

Unlike pediatric patients, where the cause is typically idiopathic, adult intussusception often involves organic pathologies. This includes both benign and malignant colonic tumors, as well as postoperative adhesions, identified as the lead point in the majority of cases [2, 3]. Intussusception initiates at this lead point, serving as a focal point of grip that pulls the proximal intestine into the distal colon [4]. In this case, we present a female patient with ileocecal intussusception as an unusual manifestation of ascending colon adenocarcinoma, which was managed through laparoscopic right hemicolectomy, representing the first reported case in Sudan.

## Case presentation

A 42-year-old Sudanese woman with a BMI of 17.4 kg/m^2^, presented with a 2-month history of severe fatigue, loss of appetite and significant weight loss, amounting to 25 kg over the last year. She experienced right upper quadrant pain that was constant, radiating to the umbilicus. Additionally, she reported intermittent diarrhea and occasional rectal bleeding over the past month, necessitating repeated blood transfusions. The patient, with no co-morbidities or smoking history, had a strong family history of colon cancer; her twin sister passed away to colon cancer three years ago.

On physical examination, the patient appeared pale and cachexic, afebrile with a pulse rate of 96/minute, a respiratory rate of 20/minute and a blood pressure of 118/80 mmHg. Abdominal examination revealed a soft, non-distended abdomen with a palpable, tender mass in the right upper quadrant region. No other palpable masses were noted. Examination of other systems did not reveal any significant findings. Digital rectal examination disclosed normal tone, no lesions, tenderness or evidence of bleeding. Laboratory findings included a hemoglobin level of 9.7 g/dL, a white blood cell count of 9.5 × 10^9^/L, and normal renal and liver function tests.

**Figure 1:**
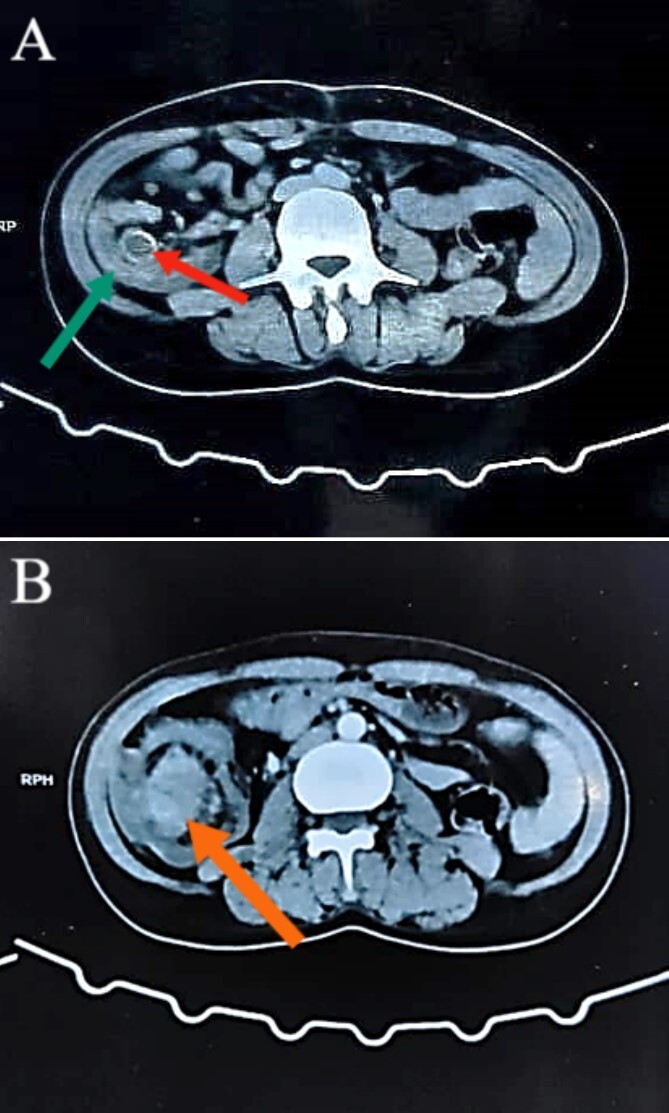
Abdominal CT images illustrating the presence of a concentric lesion in the cecum and proximal portion of the ascending colon, involving the terminal ileum. In (A), the red arrow indicates the terminal ileum within the cecal lumen, and the green arrow indicates the cecal wall (target sign). In (B), the orange arrow indicates the mass.

**Figure 2:**
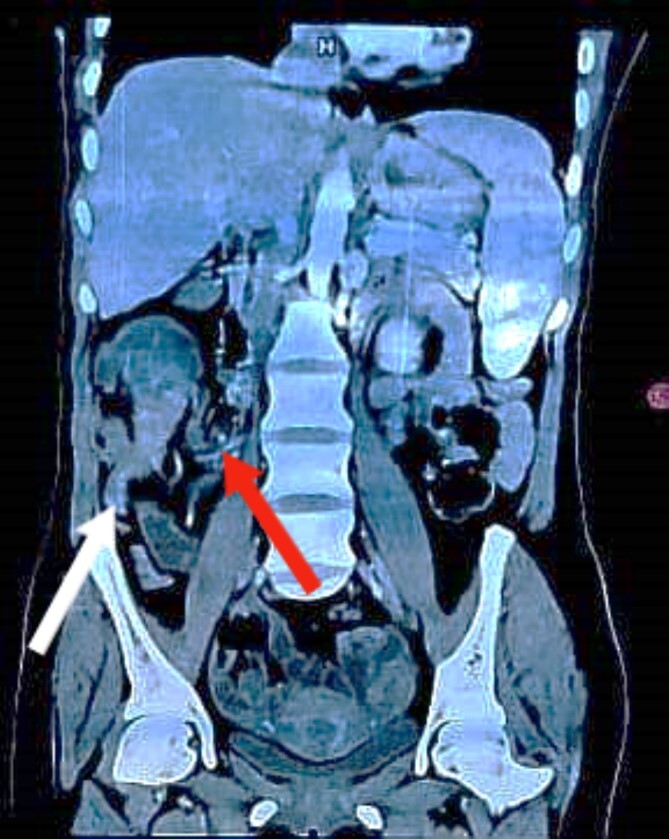
Coronal CT image demonstrating the lesion with the terminal ileum mesentery inside the cecum. The white arrow indicates the inflamed appendix. The red arrow indicates the terminal ileum and its mesentery entering the cecum.

Colonoscopy revealed a malignant-looking mass, the mass appeared irregular, circumferential, fungating and was easily bleeding. It almost occluded the lumen, which correlated with the patient’s symptoms of chronic intermittent intestinal obstruction. The histopathology report indicated invasive moderately differentiated adenocarcinoma. An abdominal CT was conducted and reported a 7 × 5 cm^2^ soft tissue mass in the ascending colon, highly suspicious for colon cancer, with features of intussusception but no evidence of metastatic disease (Figs 1 and 2). Axial and coronal CT demonstrate the lesion and the intussuscepted bowel].

**Figure 3:**
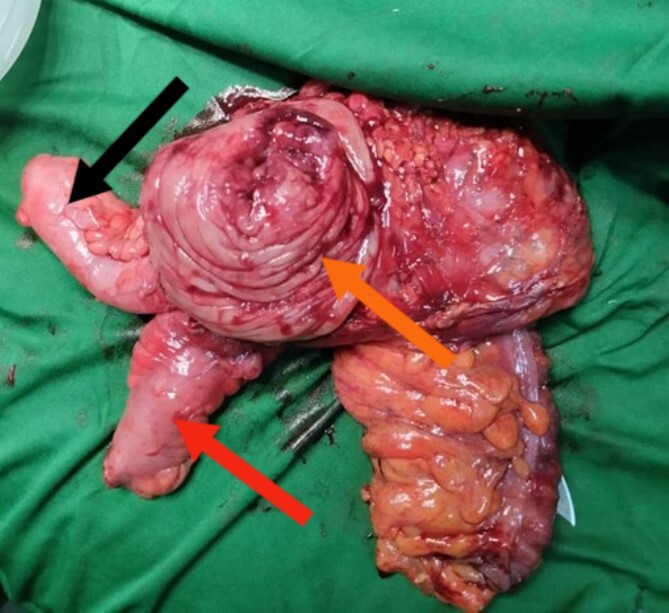
Surgical specimen post-block resection of the distal portion of the ileum and proximal portion of the ascending colon. The orange arrow indicates the mass with the intussusception. The red arrow indicates the terminal ileum. The black arrow indicates the appendix.

The laparoscopic right hemicolectomy was performed using a 10-mm supraumbilical port for open access, a 10-mm suprapubic port and a 5-mm port in the left iliac fossa. Dissection proceeded from medial to lateral, with identification and ligation of the ileocolic artery and vein, and the right branch of the middle colic artery. This was followed by complete mesocolic excision. The resected specimen, including the cecum, ascending colon and proximal third of the transverse colon, measured approximately 20 cm in length. Additionally, 15 cm of the terminal ileum was resected (Fig. 3). An extracorporeal hand-sewn ileotransverse anastomosis was performed. The back table examination confirmed the presence of a mass in the ascending colon with the terminal ileum adhering to it. The postoperative course was uneventful, and the patient was discharged on postoperative day 5. Histopathology showed that all margins were free, including the circumferential resection margin. Examination of 14 lymph nodes revealed two positives for metastasis. The mass was diagnosed as mucinous moderately differentiated adenocarcinoma. Follow-up plans included adjuvant chemotherapy as recommended by oncology. However, due to the current armed conflict in the region, the patient was unable to receive any further treatment.

## Discussion

Adult intussusception is a rare condition that can be categorized as enteric, ileocolic, colonic or ileocecal depending on its location and triggers; our patient presented with the last type [5]. Unlike children with the ‘classic’ triad of abdominal pain, vomiting and currant-jelly stools, adults with intussusception often manifest with nonspecific abdominal pain, leading to delayed diagnosis [6]. The timing of the presentation is crucial in determining patient symptoms, which can include nausea, vomiting, abdominal distension, changes in bowel habits, hematochezia and, ultimately, result in chronic intermittent intestinal obstruction [7]. In our case, weight loss, rectal bleeding and a palpable abdominal mass were present, which are indicators when intussusception coexists with malignancy.

When diagnosing adult patients with colonic or ileocolic intussusception, tumors are typically present, although clear signs of acute abdomen are mostly absent [3]. Therefore, a preoperative diagnostic approach is essential. Among preoperative diagnostic techniques, CT is the most accurate to diagnose intussusception when compared to ultrasonography, barium enema or colonoscopy [8], with the characteristic image on CT being the ‘target’-shaped sign [8]. Additionally, colonoscopy may also be used to differentiate between benign and malignant lesions through the biopsy process.

Categorizing the causes based on location reveals a high prevalence of malignant intussusception in the colonic site (46.5%). In these instances, primary colon adenocarcinoma emerged as the leading cause (78.8%), followed by lymphoma and metastasis. This underscores the significance of colonic intussusception as a potential manifestation of malignancy.

Given the elevated occurrence of primary adenocarcinoma, surgical intervention without reduction is recommended for the treatment of colonic intussusception. [8]. Right hemicolectomy without reduction was the procedure of choice in many similar cases [4, 9–11]. Total resection without reduction is performed in cases of primary adenocarcinoma to avoid potential intraluminal seeding or venous tumor dissemination [9]. As our patient was confirmed to have moderately differentiated adenocarcinoma through coloscopy biopsy, right hemicolectomy without reduction was chosen. In cases where exclusive involvement pertains to the small bowel, attempting the reduction of bowel intussusception is justified due to its lower probability of association with malignancy [9]. This shows the importance of preoperative diagnostic evaluation, as it can influence the decision of the operation. Nevertheless, exploratory laparotomy is the standard management when there are signs and symptoms of acute abdomen, and emergency laparotomy is crucial when septic shock and peritonitis are present [1].

Our patient had ileocecal intussusception as a presentation of cecal cancer. The patient, who is young, has a significant family history of colon cancer, specifically a twin sister who was diagnosed and passed away due to the cancer. There is a possibility of Lynch syndrome as an underlying cause, emphasizing the need for mandated fecal immunochemical testing every 1 to 2 years or genetic testing for MMR and MSI [12]. Unfortunately, due to the patient’s low socioeconomic status and the challenges imposed by the ongoing conflict, these tests could not be conducted. It is recommended to consider coloscopy screening for other members of the family, especially first-degree relatives.

In conclusion, this case underscores the complex presentation of adult intussusception, as it could be the first sign of underlying malignancy, therefore requiring a high index of suspicion. Here, we report the first documented Sudanese case, where ileocecal intussusception resulted from ascending colon cancer. CT scan facilitated the diagnosis, and laparoscopic right hemicolectomy provided the definitive treatment. The patient’s family history suggests a need for genetic testing, although limitations prevented it in this case. This case emphasizes the critical link between adult intussusception and cancer, highlighting the importance of considering genetic testing for patients with a family history of cancer, as it could potentially improve patient outcomes.
